# Meat Production in a Feedlot System of Zebu—Holstein Steers and Heifers with Dairy Genetics: Productive and Biological Analyses

**DOI:** 10.1155/2014/371968

**Published:** 2014-12-10

**Authors:** Gustavo Chamon de Castro Menezes, Sebastião de Campos Valadares Filho, José Reinaldo Mendes Ruas, Edenio Detmann, Arismar de Castro Menezes, Diego Zanett, Lays Débora Silva Mariz, Luciana Navajas Rennó, Jarbas Miguel da Silva Junior

**Affiliations:** ^1^Animal Science Department, Federal University of Viçosa, 36570-000 Viçosa, MG, Brazil; ^2^Agricultural Research Company of Minas Gerais (EPAMIG), Brazil

## Abstract

The aim of this study was to evaluate the productive and biological efficiency of steers and heifers from dairy genetics in a feedlot system in terms of meat production. Twenty-four steers and 24 heifers at 10 monthes of age, (3/4) Zebu × (1/4) Holstein were utilized. They were distributed over four feedlot times, 30, 60, 90, and 120 days with four replications for each sex, and were slaughtered at the end of each period. The productive and biological analyses were performed through comparative slaughter to determine the body composition. Heifers presented with greater intakes (*P* < 0.05) of dry matter in grams per kg of body weight. Steers presented with a greater (*P* < 0.05) final empty body weight, carcass gain, cold carcass weight, and meat proportion in the carcass; however, heifers presented with a greater subcutaneous fat thickness (*P* < 0.05) and, consequently, a greater (*P* < 0.05) fat proportion in the carcass. We conclude that steers are more efficient in their productive performance than heifers in a feedlot. For the finishing carcass fat cover, heifers need 90 days in the feedlot. The net energy requirements for maintenance are 67 kcal/EBW^0.75^/d, and the net requirements of energy (NEg) and protein (NPg) for gain can be estimated by the following equations: NEg(Mcal/d) = 0.067 × EBW^0.75^ × EBG^1.095^ and NPg = 162 × EBG − 5.62 × RE for the two sexes.

## 1. Introduction

To increase profitability, dairy farmers have sought to diversify production, particularly through integration with the meat chain. The reason for using beef bulls to breed dairy cattle is the increased productivity, along with improvements in the carcass and meat quality of the progeny, which result in a greater acceptance and value of calves on the market.

In the literature, strategies to define the proportion of beef bulls in dairy herd [1] and the use of specialized breed in meat production [2] are observed. However, in tropical countries, an increasing number of producers are crossing Zebu and Holstein cattle, and a lot of questions are raised regarding the productive and biological efficiency when Zebu animals crossbred with Holstein cattle are utilized for meat production in a feedlot system. The efficiency of animals can vary according to the diet, weight gain components (protein and fat deposition rates), genetic group, rate of gain, sex, and growth stage of the animals [3, 4].

In Brazil, the use of ration formulation systems that utilize net and metabolizable nutritional units is limited by the lack of information regarding the nutritional value of feedstuff and the nutritional requirements of Zebu cattle crossbred with Holstein cattle. Thus, the objective of this study was to evaluate the productive and biological characteristics of Zebu steers and heifers crossbred with Holstein cattle that are utilized for meat production in a feedlot system.

## 2. Materials and Methods

The experiment was conducted in the experimental feedlot of the Animal Science Department of the Universidade Federal de Viçosa, Viçosa, Minas Gerais, Brazil, during the period from March to August of 2012.

Twenty-four steers and 24 heifers were utilized; both sexes were (3/4) Zebu × (1/4) Holstein. They were 10 ± 2 monthes of age and had an average initial body weight of 299 ± 21.9 kg and 266.7 ± 41.6 kg, respectively. To compose the reference and maintenance groups, eight animals of each sex were randomly selected; the maintenance group was fed at 12.0 g dry matter (DM)/kg body weight. The other animals were distributed over four feedlot times (30, 60, 90, and 120 days) with four replications per sex.

The diet was formulated according to the BR-CORTE [5] for gains of 1.0 kg/d. The same diet (Table 1) was provided for all animals and consisted of 45% roughage on dry matter bases (DM) and 55% concentrate. The roughage was composed of 75% corn silage and 25% sugarcane, and the concentrate was formulated based on ground corn, soybean meal, soybean hulls, urea/ammonium sulfate, limestone, common salt, and a trace mineral mix. The diets (Table 1) were provided twice a day (all roughage in the morning), and the total amount of concentrate was provided in two equal portions at 07:00 and 14:00. The animals were weighed, identified, treated for ecto- and endoparasites, and submitted to a 21 d period for acclimation to the experimental conditions, after which the animals from the reference group were slaughtered.

The relationship between the empty body weight (EBW) and the shrunk body weight (SBW) was utilized to estimate the initial EBW of the remaining animals and the relationship between the carcass weight and the body weight of the reference animals was calculated to estimate the initial carcass weight of the remaining animals in the experiment. The amount of ration offered per animal as well as the ort was recorded daily and was sampled, after which a composite sample was performed for each 30 d period.

The animals were weighed every 30 days. The experiment was divided into 4 periods of 30 days, with the animals slaughtered at the end of each period (30, 60, 90, and 120 days). The animals were kept in a tie stall system in covered pens with a concrete floor, automatic drinker, and individual feeders. Digestibility assays were performed before each slaughter period, where we collected the total amount of feces that were excreted over three consecutive days. When the 24 h period of collection was completed, the feces were weighed and homogenized, and a sample was extracted. Feces and feed samples were dried in a forced oven at 55°C for 72 hours, after which they were ground in a knife mill with a 1 mm screen. Samples of feed and feces were analyzed for the contents of dry matter (DM), organic matter (OM), and crude protein (CP) that were evaluated according to the methods described by [6]. The ether extract (EE) was quantified using Soxhlet extraction with petroleum ether. The neutral detergent fiber (NDF) content was estimated according to Mertens [7] and NDF was corrected for ash [8] and for protein according to [9]. The analyses of NDF were performed by using a fiber analyser (Ankom200, Ankom Technology, Macedon, NY, USA). The nonfiber carbohydrates (NFC) were calculated as proposed by [10]: NFC = 100 − [(%CP − %CP from urea + %urea) + %apNDF + %EE + %ash], where NFC is nonfiber carbohydrates, CP is crude protein, apNDF is neutral detergent fiber corrected for protein and ash, and EE is ether extract. The total digestible nutrients (TDN) of the diets were estimated through the sum of the digestible nutrients. The digestible energy intake (DEI) was obtained by multiplying the digestible nutrients with their respective energy values as described by [8]: DEI = 5.6 × DCPI + 9.4 × DEEI + 4.2 × DapNDFI + 4.2 × DNFCI, where D before the nutritional variable means digestible and I after the variables means intake. The metabolizable energy (ME) concentration was considered to be 82% DE [11].

At the end of each 30 d period, eight animals, four of each sex, were slaughtered while following the Humanitarian Slaughter Standards approved by the Ethics Committee of UFV, process number 07/2013. Prior to the slaughter, the animals were submitted to a 16 h solid fasting period. The slaughter was performed through brain concussion and total bleeding via jugular section, followed by washing of the gut tract. Heart, lungs, spleen, kidney, KPH fat, diaphragm, mesentery, tail, trachea, esophagus, reproductive trat, and gut tract (after washing), head, hide, limbs, blood, and carcass were weighed for evaluation of EBW. After the slaughter, the carcass of each animal was divided into two halves that were then weighed for evaluation of the hot carcass yield and were then cooled in a cold chamber at 4°C for 24 hours. After the cooling period, the carcasses were weighed for evaluation of the cold carcass yield, while also measuring the carcass length. All right half-carcass sections were dissected for bone, muscle, and fat, after which these components were ground and a composite sample was assembled proportional to its amount in the empty body weight.

The rumen, reticulum, omasum, abomasum, small and large intestines, KPH fat, mesentery, liver, heart, kidney, lungs, tongue, spleen, diaphragm, esophagus, trachea, and reproductive tract were ground in an industrial cutter to create a composed and homogenized sample of organs and viscera. The blood was collected after total bleeding and was packed in a plastic container. In addition, the head and limbs were ground in an industrial cutter and the hide was cut. These components were sampled, and a composite sample of noncarcass components was assembled proportionally to the empty body weight of each animal. These samples were obtained for each animal and were lyophilized for 72 hours to quantify the partial fat dry matter. After that, these samples were partially defatted through successive washes with petroleum ether. After the partial defatting, the samples were ground in a knife mill to quantify the contents of DM, ash, CP, and EE as previously described. The samples of the carcass and noncarcass components together comprised the chemical composition of the empty body of the animal.

To estimate the body composition in terms of CP, ash, and water, the EBW was utilized in the allometric model: *C*_*i*_ = *α* × EBW^*β*^, where *C*_*i*_ is body component “*i*” of the animal defined as CP, ash, or water present in the empty body (kg) and “*α*” and “*β*” are regression parameters. The EE present in the empty body was estimated by the exponential model: *C*_*i*_ = *a* × *e* ^(*b*×EBW)^, where *C*_*i*_ = EE in the animal's body (kg) and *e* is Euler number. The FE (feed efficiency) was obtained by dividing the weight gain by DMI in kg.

Data for intake, nutrient digestibility, and performance were analyzed according to a completely randomized design in a 2 × 4 factorial scheme composed of sex (steers and heifers) and four feedlot periods (30, 60, 90, and 120 days); the effects of sex and period as well as the interaction between these two variables were also evaluated. The feedlot time was evaluated as linear, quadratic, and cubic contrasts by using the PROC MIXED component of the SAS program (version 9.3). The statistical analysis of the biological effect was performed through multiple linear regressions by using the model RP = *β*1EBG + *β*2(EBG∗*D*) + *β*3RE + *β*4(RE∗*D*) + *ϵ*, where *D* was the dummy variable (binary) of the effect of sex (*D* = 0 for heifer and *D* = 1 for steer).

## 3. Results and Discussion

### 3.1. Intake and Nutrient Digestibility

The intake in kg/d of DM, OM, CP, EE, digestible organic matter (DOM), TDN, and DE did not demonstrate an effect (*P* > 0.05) of interaction between sex and feedlot periods. However, when the intake was expressed in grams per kg of body weight (BW), an effect was observed (*P* < 0.05) for sex and period on the intake of DM, OM, and apNDF, with greater values for the heifers (*P* < 0.05) (Table 2). Cubic effects were observed for the apNDF intake when expressed as kg/d and in g/BW. There was no observed effect for the interaction between sex and period or an effect of sex on the digestibility of OM, CP, apNDF, and EE. However, the OM digestibility increased while the apNDF digestibility decreased linearly in association with the increased feedlot period. The increase in OM digestibility can be explained by the reduction in the intake in grams per kg of body weight as the feedlot time increased, because the digestibility is the result between digestion and passage rates, which are positively correlated with dry matter intake [1].

The study of [12] reported that heifers with a body weight lower than 250 kg presented with a greater capacity for intake than bulls and steers. Due to the fact that the heifers present with a greater body fat deposition, the capacity for intake should decrease because the fat exerts a direct influence due to physical limitations provided by the abdominal fat on the rumen and an indirect influence on feed intake through the secretion of leptin by adipocytes, a hormone that has been correlated with a reduction in intake [13]. However, the heifers were slaughtered at a younger age, which may justify the greater intake. A difference was not observed (*P* > 0.05) in FE among sexes.

Steers presented with a greater (*P* < 0.05) fEBW, CG, CCW, and proportion of meat in the carcass; however, heifers finished with a greater SFT (*P* < 0.05) and consequently a greater (*P* < 0.05) proportion of fat in the carcass (Table 3). The effect of feedlot time on fEBW, CCW, SFT, and CY demonstrated that 120 days in the feedlot results in greater (*P* < 0.05) values for the first three variables, while the CY was lower (*P* < 0.05) at 30 days in the feedlot for the two sexes. The quadratic effect (*P* < 0.05) observed for CG and proportion of fat in the carcass shows that greater gains were made with increasing feedlot time.

Steers and heifers presented with a precocity towards fat deposition in the carcass at 90 days in the feedlot. In addition, the subcutaneous fat thickness has a protective function for the carcass against the effects of cooling to avoid darkening of the meat. Several genetic and ambient factors such as genetic group, age, sex, and nutritional level influence the standard tissue deposition and body components and consequently the body composition of beef cattle [14]. Steers presented with a greater (*P* < 0.05) proportion of meat in the carcass, while heifers presented with a greater (*P* < 0.05) proportion of fat. Steers and heifers did not present with differences (*P* > 0.05) in terms of losses during the cooling period or in carcass length.

Steers and heifers presented a carcass with moderate final weights but obtained a higher carcass yield when compared to the results of [15, 16]. These authors did not find a difference in carcass yield in the progeny of Holstein cows bred with British beef bulls, Angus (CY 48.4% and carcass weight of 200 kg), Devon (CY 48.4% and carcass weight of 214 kg), or South Devon (CY 50% and carcass weight of 253 kg).

An interaction was observed (*P* < 0.05) between sex and the feedlot periods in terms of the average daily gain and the empty body gain (Table 4). Steers presented with a greater (*P* < 0.05) average daily gain and empty body gain. The lower growth rate of heifers is possibly due to a greater participation of the KPH fat, whereas the fat deposition in the abdomen occurs at the expense of weight gain [17].

The performance of animals with dairy origins can be considered adequate for meat production in a feedlot system when compared to the performance results of 1.28 kg/d obtained by [18] for crossbred Holstein × Zebu bulls that were fed a diet based on sugarcane. However, the main point is with regard to the animal category and physiological factor, because the animals are from dairy origins, and prior to the feedlot, their feed was composed of a liquid diet and tropical pasture; even according to their age and slaughter weight, these animals are classified as early maturity. The intensification of the production system by slaughtering young animals results in a better finishing carcass and better meat quality, thereby contributing to greater values in the market. Thus, the use of dairy animals in a feedlot system for meat production is an interesting option due to the fact that these animals have a lower commercial value when compared to beef breeds.

The high subcutaneous fat thickness and percentage of fat in the carcass at 120 days showed that heifers needed 90 days in the feedlot for the carcass finishing period, which is important to consider in terms of the economic effects of the commercialization of carcasses with lower body weights.

### 3.2. Biological Effect

In Table 5, the chemical composition of the empty body of the animal is shown. There was no observed effect of sex, and the following equations were utilized to predict the carcass components: EE = 0.0156 × *e* ^(1.4295×EBW)^; CP = 0.1782 × EBW^0.9782^; ash = 0.2199 × EBW^0.6141^; and water = 1.1620 × EBW^0.8937^.

The equation that was obtained for steers and heifers for the relationship between empty body weight (EBW) and shrunk body weight (SBW) in kg was as follows: EBW = 0.898 × SBW. Related to performance of the animals, an equation was obtained between empty body gain (EBG) and average daily gain (ADG) in kg, where EBG = 0.951 × ADG. The relationship between EBW and EBG with regard to the shrunk body weight and average daily gain was similar to those described by [4, 11, 19].

The heat production (HP) was measured indirectly by determining the difference between metabolizable energy intake (MEI) and retained energy (RE) in the empty body of the animal; it did not differ among sexes and was obtained through the following equation: HP = 0.0669 × *e* ^4.2306×MEI^.

In meat production, the nutritional costs for maintenance are important components of the total production costs. As the costs for maintenance are closely associated with body weight, smaller animals possibly have lower requirements for body maintenance. The net energy requirement for maintenance (NEm) was approximately 67 kcal/kg EBW^0.75^ for both sexes. The requirements that were demonstrated for the animals are similar to the experimental observations of [4] when using bulls of the same genetics, nutritional conditions, and handling. In the literature, values of 77 and 74.2 kcal/kg EBW^0.75^ were presented by [5, 11], respectively, and were obtained from animals that were crossed with Nellore or* Bos taurus* beef cattle.

**Figure 1:**
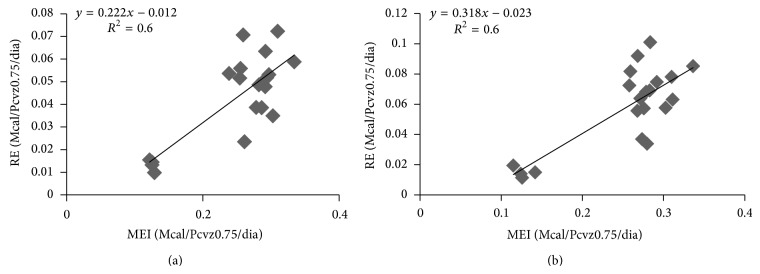
Retained energy (RE) as a function of the metabolizable energy intake (MEI) of steers (a) and heifers (b).

The metabolizable energy requirement for maintenance (MEm) obtained by the iterative method equated HP to MEI in the equation above. The EMm was approximately 104 kcal/kg EBW^0.75^ for both sexes, which is close to those reported by [5]. The efficiency of the use of metabolizable energy for maintenance (km) was obtained by dividing Nem by Mem to obtain 0.64 for the animals, which was also observed by [20, 21]. The equation for RE was adjusted as a function of EBW and EBG to obtain the net energy requirements for gain for any range of body weight and weight gain. Steers and heifers did not demonstrate differences in the parameters of the equations; the equation that was obtained for both sexes is NEg = 0.067 × EBW^0.75^  × EBG^1.095^, where NEg is the net energy requirements for gain (Mcal/d), EBW^0.75^ is metabolic empty body weight in kg, and EBG is empty body gain in kg. The intercept of the equation is the average of the values presented by [19]. To convert the net energy requirements to metabolizable energy requirements for gain, it is necessary to know the efficiency of the use of metabolizable energy for weight gain (kg). This efficiency was obtained through the regression between RE and MEI (Figure 1).

The efficiency of the use of ME for gain was influenced by sex; the values that were obtained for the efficiencies were 0.22 for steers and 0.32 for heifers. The efficiency obtained for heifers is similar to the value of 0.33 reported by [4]. The efficiencies of protein and fat are different as a function of the proportions for gain of each component in the animal's body [12]. The equation that was obtained to generate the efficiency values for both sexes was MEI = 0.1035 + 1.40 × REf + 6.01 × REp, where MEI is metabolizable energy intake (Mcal/kg EBW^0.75^/d), while REf and REp are the energies retained as fat and protein in Mcal/kg EBW^0.75^/d. The variable efficiencies of fat and protein deposition were obtained through the inverse of the estimated coefficients; the values that were obtained were 0.71 (*k*_fat_) and 0.17 (*k*_protein_) for steers and heifers. Greater efficiency values of 0.83 for fat and 0.34 for protein were obtained by CHIZZOTTI [22]. The carcass characteristics presented in Table 3 show concordance of the factors of efficiency for fat deposition in relation to protein deposition in the carcass because of an increased effect in proportion for fat and decrease for protein.

With the increase in body weight, there occurred a decrease in the proportion of protein and an increase in the proportion of fat in the empty body weight due to the reduction of the muscle growth and an increase in the development of adipose tissue [23]. As a consequence, the energy requirements increased, while the protein requirements decreased.

**Figure 2:**
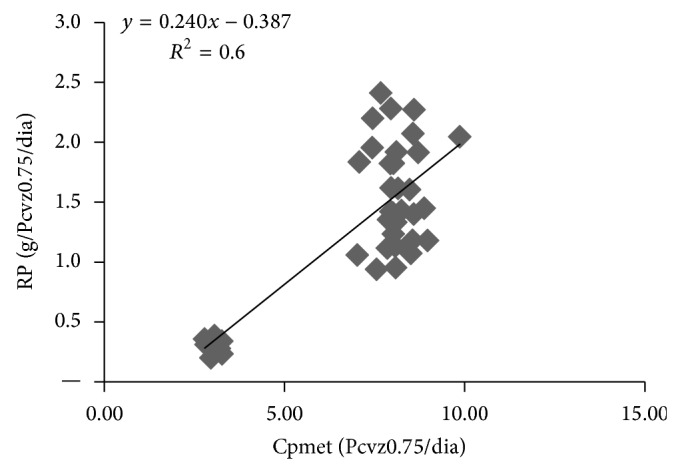
Retained protein (RP) as a function of the metabolizable protein intake (Cpmet) of steers and heifers.

The protein requirements for maintenance for both sexes were approximately 3.0 grams/EBW^0.75^. Based on the equation adjusted for retained protein (RP) as a function of the metabolizable protein intake (MPI), the coefficient of inclination was 24.5% for steers and heifers if we consider the efficiency of use of metabolizable protein for gain shown in Figure 2. The values that were obtained are below the 37% observed by [4] and the 46.7% recognized by [5].

The net protein requirements for gain (NPg) were estimated from the RP as a function of retained energy (RE) and empty body gain (EBG). There was no effect of sex when NPg was related to EBG, which was obtained through the following equation: NPg = 162 × EBG − 5.62 × RE. The authors [24] demonstrated that steers present with greater net protein requirements for gain than heifers since there was a greater deposition of lean tissue in the bodies of animals of the same age, a fact that was not observed in this experiment.

## 4. Conclusions

Steers are more efficient at gaining weight than crossbred (3/4) Zebu × (1/4) Holstein heifers finished in a feedlot system. Heifers presented with a greater dry matter intake in grams per kg of body weight in relation to steers. For carcass finishing, in order to obtain a fat cover of 5 mm, heifers need 90 days in the feedlot. The net energy requirements for maintenance of steers and heifers are 67 kcal/EBW^0.75^ and the net requirements of energy (NEg) and protein (NPg) for gain can be estimated by the following equations: NEg = 0.067 × EBW^0.75^  × EBG^1.095^ and NPg = 162 × EBG − 5.62 × RE. The efficiencies of energy deposition as fat and protein for steers and heifers are 71% and 17%. Steers and heifers of dairy origins are efficient in performance and are an option for producing meat in a feedlot system.

## Figures and Tables

**Table 1:** Proportions of feed in the concentrate and diet and the composition of concentrates and diets on a dry matter basis.

Ingredients	Concentrate	Diet
	Proportion (% DM)	
Corn silage	—	33.7
Sugarcane	—	11.2
Soybean hulls	54.5	30.0
Ground corn	36.0	19.8
Soybean meal	6.0	3.3
Urea/ammonium sulfate	1.8	1.0
Common salt	0.8	0.5
Limestone	0.7	0.4

	Chemical composition (% DM)	
Dry matter	89.1	63.5
Organic matter	97.1	96.8
Crude protein	16.9	11.6
Neutral detergent fiber^1^	38.2	44.7
Nonfiber carbohydrates	42.1	40.0
Ether extract	2.6	2.0

^1^Corrected for ash and protein.

**Table 2:** Intake in kg and g/kg live weight and nutrient digestibility in % of DM intake of steers and heifers with dairy origins in a feedlot.

Item	Sex		Period (days)				RSD	*P* value					
	S	H	30	60	90	120		Sex	Period	S∗P	LIN	Quad	CUB
Intake (kg/day)													
DM	8.18	7.55	7.59	8.02	7.60	8.27	0.88	0.058	0.366	0.619	0.278	0.705	0.177
OM	7.87	7.27	7.32	7.72	7.29	7.95	0.85	0.058	0.364	0.619	0.300	0.667	0.169
CP	0.67	0.62	0.61	0.66	0.63	0.69	0.07	0.067	0.181	0.633	0.109	0.755	0.129
apNDF	2.05	1.87	2.13	2.23	2.22	1.26	0.22	0.040	<0.001	0.480	<0.001	<0.001	<0.001
EE	0.04	0.05	0.05	0.05	0.05	0.05	0.06	0.387	0.651	0.550	0.214	0.454	0.577
DOM^1^	5.48	5.01	4.87	5.39	5.08	5.65	0.65	0.054	0.133	0.554	0.069	0.928	0.116
TDNI	5.54	5.05	4.92	5.44	5.13	5.69	0.50	0.058	0.364	0.619	0.300	0.0339	0.166
DEI^*^	24.3	22.2	21.7	23.9	22.5	25.0	2.88	0.051	0.140	0.556	0.079	0.900	0.110
MEI^*^	18.5	18.2	17.8	19.6	15.5	20.5	3.85	0.831	0.046	0.338	0.519	0.255	0.015
FE^*^	0.12	0.11	0.16	0.10	0.11	0.10	1.51	0.190	<0.001	0.205	0.003	0.016	0.003

Intake (g/kg live weight)													
DM	22.4	23.9	25.3	23.8	22.8	20.8	1.40	<0.001	<0.001	0.202	<0.001	0.607	0.455
OM	21.5	23.0	24.3	22.8	21.9	20.0	1.34	<0.001	<0.001	0.204	<0.001	0.692	0.487
apNDF	5.70	6.07	7.09	6.61	6.67	3.17	0.36	<0.001	<0.001	0.105	<0.001	<0.001	<0.001

Digestibility (% DM intake)													
OM	69.6	68.8	66.4	69.7	69.6	70.9	2.58	0.418	<0.001	0.813	0.005	0.295	0.239
CP	66.2	63.0	65.9	65.5	61.1	65.9	5.12	0.097	0.198	0.825	0.623	0.166	0.113
apNDF	44.1	40.6	45.1	43.6	44.6	36.1	7.02	0.172	0.062	0.519	<0.001	0.186	0.285
EE	70.48	69.71	68.03	70.79	69.53	72.04	0.96	0.058	0.364	0.619	0.0240	0.0094	0.481

Sex (S = steer, H = heifer); ^1^DOM = digestible organic matter obtained through multiplication of intake and its digestibility; RSD = residual standard deviation; S∗P = effect of the interaction between sex and period; LIN = linear effect; QUAD = quadratic effect; CUB = cubic effect; ^*^DEI and MEI in Mcal/d; FE = feed efficiency.

**Table 3:** Carcass characteristics of steers and heifers with dairy origins finished in a feedlot.

Item	Sex		Period (d)				RSD	*P* value					
	S	H	30	60	90	120		Sex	Period	S∗P	LIN	QUAD	CUB
fEBW/kg	330	287	261	308	303	361	29.7	<0.001	<0.001	0.467	<0.001	<0.001	<0.001
CG/kg	72	61	32	55	79	100	9.84	<0.001	<0.001	0.066	<0.0001	<0.0001	0.889
CCW/kg	203	176	164	187	182	225	17.51	<0.001	<0.001	0.134	<0.001	<0.001	<0.001
SFT mm	4.63	5.96	3.38	5.41	5.06	7.34	1.47	0.0249	<0.001	0.331	<0.001	<0.0001	0.006
CL %	1.99	2.12	2.12	2.11	2.04	1.95	0.38	0.339	0.785	0.317	0.339	0.376	0.947
CY/kg	56	55	53	57	56	57	1.27	0.279	<0.001	0.860	<0.001	<0.001	<0.001
Length/cm	121	122	122	122	119	123	7.24	0.665	0.550	0.525	0.859	0.861	0.269
Meat %	58	55	59	56	57	54.1	2.53	<0.001	<0.001	0.872	<0.001	<0.001	0.080
Fat %	19	23	18	21	21	26.0	3.07	<0.001	<0.001	0.447	<0.001	<0.001	0.163
Bone %	18	17	19	17	17	16	2.04	0.149	0.034	0.552	<0.001	<0.001	0.143

^*^Sex (S = steer, H = heifer); fEBW = final empty body weight in kg; CG = carcass gain in kg; CCW = cold carcass weight after cooling for 24 h at 4°C; SFT = subcutaneous fat thickness in the carcass; CL = cooling losses/% of carcass; CY = carcass yield; length = carcass length in cm; meat, fat, and bone are the proportion of the components in relation to the cold carcass; RVC = residual variable covariance; S∗P = effect of interaction between sex and period; LIN = linear effect; QUAD = quadratic effect; CUB = cubic effect; RSD = residual standard deviation.

**Table 4:** Interaction between sex and period on the average daily gain and empty body gain (kg/d) of steers and heifers with dairy origins in a feedlot.

Sex	Period				RSD	*P* value		
	30	60	90	120		LIN	QUAD	CUB
Average daily gain								
Steer	1.49^a^	0.84^a^	1.00^a^	0.84^a^	0.15	<0.001	<0.001	<0.001
Heifer	1.03^b^	0.76^a^	0.73^b^	0.83^a^	0.14	0.068	0.002	0.723
*P* value	<0.001	0.413	0.0017	0.887				

Empty body gain								
Steer	1.25^a^	0.99^a^	1.02^a^	0.82^a^	0.11	<0.001	0.306	0.678
Heifer	0.85^b^	0.85^a^	0.72^b^	0.83^a^	0.13	0.055	0.012	0.543
*P* value	<0.001	0.341	<0.001	0.211				

SRD = standard error of mean; S∗P = effect of interaction between sex and period; LIN = linear effect; QUAD = quadratic effect; CUB = cubic effect.

**Table 5:** Chemical composition (%) of steers and heifers from dairy origins finished in a feedlot, expressed in percentage of the carcasses.

	Days in feedlot (steer)						Days in feedlot (heifer)					
	0	30	60	90	120	Maintenance	0	30	60	90	120	Maintenance
Chemical composition of the empty body weight (%)												
CP	12.67	14.59	16.64	17.50	22.09	16.16	13.42	16.79	20.90	20.52	25.09	16.25
EE	16.29	15.18	15.91	15.60	15.56	17.10	16.52	15.91	15.67	15.73	15.23	16.76
Ash	3.32	2.26	2.01	2.56	2.10	2.57	3.22	2.37	2.40	2.07	2.45	2.88
Water	67.72	67.97	65.44	64.34	60.25	64.17	66.85	64.94	61.03	61.68	57.23	63.12

^*^CP: crude protein; EE: ether extract; ash and water are the proportion in percentage of the components in carcasses.
